# Selection of reference genes for quantitative real-time PCR expression studies in the apomictic and sexual grass *Brachiaria brizantha*

**DOI:** 10.1186/1471-2229-9-84

**Published:** 2009-07-02

**Authors:** Érica Duarte Silveira, Márcio Alves-Ferreira, Larissa Arrais Guimarães, Felipe Rodrigues da Silva, Vera Tavares de Campos Carneiro

**Affiliations:** 1 Embrapa Genetic Resources and Biotechnology, Parque Estação Biológica, PqEB Av. W5 Norte (final) Caixa Postal 02372, Brasília, Brasil; 2 Department of Genetics, Federal University of Rio de Janeiro Av. Prof. Rodolpho Paulo Rocco, s/n Prédio do CCS Instituto de Biologia, 2o Andar – Rio de Janeiro, RJ, Brasil

## Abstract

**Background:**

*Brachiaria brizantha *is an important forage grass. The occurrence of both apomictic and sexual reproduction within *Brachiaria *makes it an interesting system for understanding the molecular pathways involved in both modes of reproduction. Quantitative real time PCR (qRT-PCR) has emerged as an important technique to compare expression profile of target genes and, in order to obtain reliable results, it is important to have suitable reference genes. In this work, we evaluated eight potential reference genes for *B. brizantha *qRT-PCR experiments, isolated from cDNA ovary libraries. Vegetative and reproductive tissues of apomictic and sexual *B. brizantha *were tested to validate the reference genes, including the female gametophyte, where differences in the expression profile between sexual and apomictic plants must occur.

**Results:**

Eight genes were selected from a cDNA library of ovaries of *B. brizantha *considering the similarity to reference genes: EF1 (elongation factor 1 alpha), E1F4A (eukaryotic initiation factor 4A), GAPDH (glucose-6-phosphate dehydrogenase), GDP (glyceroldehyde-3-phosphate dehydrogenase), SUCOA (succinyl-CoA ligase), TUB (tubulin), UBCE (ubiquitin conjugating enzyme), UBI (ubiquitin). For the analysis, total RNA was extracted from 22 samples and raw Ct data after qRT-PCR reaction was analyzed for primer efficiency and for an overall analysis of Ct range among the different samples. Elongation factor 1 alpha showed the highest expression levels, whereas succinyl-CoA ligase showed the lowest within the chosen set of samples. GeNorm application was used for evaluation of the best reference genes, and according to that, the least stable genes, with the highest M values were tubulin and succinyl-CoA ligase and the most stable ones, with the lowest M values were elongation factor 1 alpha and ubiquitin conjugating enzyme, when both reproductive and vegetative samples were tested. For ovaries and spikelets of both sexual and apomictic *B. brizantha *the genes with the lowest M values were *Bbriz*UBCE, *Bbriz*E1F4A and *Bbriz*EF1.

**Conclusion:**

In total, eight genes belonging to different cellular processes were tested. Out of them, *Bbriz*TUB was the less stable while *Bbriz*EF1 followed by *Bbriz*UBCE were the more stable genes considering male and female reproductive tissues, spikelets, roots and leaves. Regarding the best reference genes for ovary tissues, where apomictic and sexual reproduction must occur, the best reference genes were *Bbriz*UBCE, *Bbriz*E1F4A and *Bbriz*EF1. Our results provide crucial information for transcriptional analysis in the *Brachiaria *ssp, helping to improve the quality of gene expression data in these species, which constitute an excellent plant system for the study of apomixis.

## Background

*Brachiaria *is an important *Poaceae *genus introduced in Brazil from Africa. This genus consists of around 100 species, and the two most important cultivars in Brazil are *B. brizantha *cv. Marandu and *B. decumbens *cv. Basilisk [1]. They show qualities of forage grass, good adaptability to cerrado areas (dry-tropical savanna, Brazil), and are cultivated in more than 40 million hectares in Brazil [2]. Both cultivars reproduce asexually through seeds by apomixis [3], which is classified as a pseudogamous aposporic type [4-9]. Apomixis is present in more than 300 angiosperm species [10] and is being investigated by many groups due to the biotechnological interest of controlling the process of cloning through seeds.

The occurrence of both apomictic and sexual reproduction within *Brachiaria *makes it an interesting system for understanding the molecular pathways involved in both modes of reproduction. The identification of genes involved in apomictic development will open the possibility of controlling the expression of this trait and engineering crops with higher productivity and a reduced risk of gene transfer. One way of comparing these different molecular pathways is by comparing the transcript expression profiles of genes related to ovary development in sexual plants, which have a Polygonum-type embryo sac, to an apomictic plant, which has a Panicum-type embryo sac [9]. Analysis of a *Brachiaria *germplasm collection assembled at CIAT-Colombia pointed to a majority of polyploids apomicts, whereas the diploids are sexual [3,11]. In *B. brizantha *among 275 accessions identified to date only one is diploid, BRA 002747 [3]. Sexual tetraploids were obtained with colchicine treatment of the diploid plants [12,13]. These plants are under analysis at the breeding program aiming to produce intraspecific hybrids and to identify molecular markers associated with the apomixis trait. Currently, comparative studies of the molecular biology of *Brachiaria *reproductive processes are being performed with BRA 002747 and BRA 00591 [13,14]. Both accessions are very important for these comparative studies since the sexual diploid BRA 002747 is the only sexual accession among all the accessions, while BRA 00591 is the most apomictic accession, with 98% of aposporous embryo sacs [9].

Quantitative real-time PCR (qRT-PCR) has emerged as an important technique to compare the expression profiles of target genes in different species, tissues or treatments and also to validate high-throughput gene expression profiles [15,16]. One of the methodologies to determine gene expression levels in qRT-PCR is by comparing the expression of the gene of interest in different conditions with reference genes whose expressions do not change under the various experimental conditions. Based on these requirements, statistical analysis methods have been developed in order to identify the best reference genes to a certain organism or experimental condition [17-19]. The use of reference genes without prior verification of their expression stability can lead to inaccurate data interpretation and thus generate incorrect results.

According to previous work concerning the best reference genes for transcription normalization in plants, the most reliable ones are those constitutively expressed and involved in basic cellular processes, such as protein and sugar metabolism and cell structure [18,20-22]. A large-scale comparative analysis of the most stable genes of *Arabidopsis *has shown that the best reference genes are those related to the ubiquitin degradation process, such as polyubiquitin, ubiquitin-conjugating enzymes and ubiquitin ligases [23]. In the qRT-PCR expression profile analysis of suitable reference genes for poplar (*Populus trichocarpa *× *P. deltoides*, cottonwood hybrid) and vitis (*Vitis vinifera*), tubulin and actin were stably expressed and considered the most reliable ones [18,22]. In a similar approach, Jain et al. (2006) showed that the best genes among the different tested tissue samples in *Oryza sativa *were ubiquitin 5 and elongation factor-1 alpha. For species with both sexual and apomictic reproductive mode, the best reference genes for qRT-PCR experiments have not been reported yet. Real time PCR has been done to validate other differential expression experiments using absolute qRT-PCR or using internal control genes tested by other differential expression techniques [24,25].

In this work, we evaluated eight potential reference genes isolated from EST ovary libraries for *Brachiaria brizantha *qRT-PCR experiments. Vegetative and reproductive tissues of apomictic and sexual *B. brizantha *were tested. The relative transcription levels of the genes were determined in ovaries and anthers at different developmental stages, sporogenesis and gametogenesis, in spikelets, leaves and roots all together. Also, it was determined the most stable genes only for spikelets and ovaries, where differences in the expression profile between sexual and apomictic plants must occur, from both sexual and apomictic accessions.

## Results and discussion

### Candidate reference genes

In order to pinpoint the best reference genes in *Brachiaria*, known reference genes from other species were used to BLAST search against a *Brachiaria brizantha *EST (expressed sequence tag) library constructed from ovaries of apomictic plants in megasporogenesis and megagametogenesis. This library was validated by sequencing and annotating 2,000 clones, and out of these sequences, eight genes were chosen due to their high similarity to sequences related to commonly used reference genes, including polyubiquitin, ubiquitin-conjugating enzymes, elongation factor-1 alpha, glyceraldehyde-3-phosphate dehydrogenase and tubulin. Specific primers were designed and tested for amplification efficiency, including two sets of primers for an ubiquitin-conjugating enzyme to use as an internal control (Table 1).

**Table 1:** Gene description, primer sequences and efficiency of the selected ESTs.

***Gene identification/Gene description***	***E value/ID (%)***	***Primer sequence/Amplicon size***	Amplification efficiency ± SD*	***GeneBank Accession Number***
***Bbriz*EF1** Elongation factor-1 alpha	4e-89/ 166/179 (92%)	5'ACCCTCCTCTTGGTCGTTTT3' 5'AGCCCCTCATTTCTTCTTGG 3' 105 bp	0.87 ± 0.012	EZ000623
***Bbriz*EIF4A** Eukaryotic initiation factor 4A	4e-41/ 88/100 (88%)	5'TAAGGTGGGGCTTGTTTTTG3' 5'ACAGCAGCACATACCACAGG3' 164 bp	0.94 ± 0.011	EZ000622
***Bbriz*GAPDH** glucose-6-phosphate dehydrogenase	2e-39/ 86/121 (71%)	5'TGAATCTAGTCCATCCGCTTG3' 5'TCATCAGGCAGGGAAGCTA3' 124 bp	0.97 ± 0.009	GE617483
***Bbriz*GDP** glyceroldehyde-3-phosphate dehydrogenase	6e-22/ 48/55(87%)	5'GGGCATTTTGGGTTATGTTG3' 5'TCCCCACTCGTTGTCATACC3' 146 bp	1.01 ± 0.009	EZ000624
***Bbriz*SUCOA** succinyl-CoA ligase (GDP-forming) beta-chain	e-107/ 203/236 (86%)	5'CAGCAAGGGAGGAACCAGTA3' 5'TAGCGCAAGACCATCAACAA3' 130 bp	1.00 ± 0.008	GE617476
***Bbriz*TUB** putative tubulin alpha-5 chain	4e-51/ 96/98 (97%)	5'ATGAAGGCGATGAAGGAGAA3' 5'GTACGCAATGGAATGGAACC3' 112 bp	1.01 ± 0.019	GE617477
***Bbriz*UBCE1** Ubiquitin-conjugating enzyme ***Bbriz*UBCE2**	4e-31/ 64/74 (86%)	5'GGTCTTGCTCTCCATCTGCT3' 5'CGGGCTGTCGTCTCATACTT3' 114 bp 5'ACCAGCACAAATCAAAGGA3' 5'GCCAAAGTATGAGACGACAGC3' 149 bp	0.92 ± 0.013 0.95 ± 0.015	GE617481
***Bbriz*UBI** ubiquitin/ribosomal protein	4e-06/ 28/49 (57%)	5'GTCACTAAGCCATCGGTCGT3' 5'ACACGGACACAACCAGTTCA3' 112 bp	0.94 ± 0.020	GE617482

*Amplification efficiency was calculated using the miner algorithm [21] and range from 0.5 (50%) to 1.5 (150%).

### Primer efficiency and Ct variation

In order to find the best reference genes for relative quantification, a high quality starting material is needed. For that, total RNA was extracted from all tissue samples using the same extraction protocol [14] for the different *Brachiaria *organs. All samples were treated with DNAse to avoid misinterpretation of qRT-PCR results by genomic DNA contamination in cDNA samples. RNA quality analysis and quantitation were performed by agarose gel analysis and a Nano-Drop ND-1000 spectrophotometer (NanoDrop Technologies) measurement, respectively. This procedure was crucial to guaranteeing the same amount of starting material and equivalent efficiency of cDNA synthesis from total RNA samples.

Based on DNA analysis by agarose gel electrophoresis and the dissociation curves (additional file 1), one single PCR product with the expected size was amplified for each of the nine sets of primers selected for this analysis (not shown). After the PCR reaction, the entire raw fluorescence data generated in Opticon3 was used for the primer amplification efficiency calculation and Ct determination with the miner algorithm [26]. This algorithm accounts for each PCR exponential curve, making it is possible to have accurate values for the quantification of qRT-PCR. The amplification efficiency using this program can vary between 50% and 150%, and for the nine tested primer pairs it varied from 0.87 ± 0.012 (87%) to 1.01 ± 0.009 (101%), which are expected amplification efficiencies between compared genes [26].

**Figure 1:**
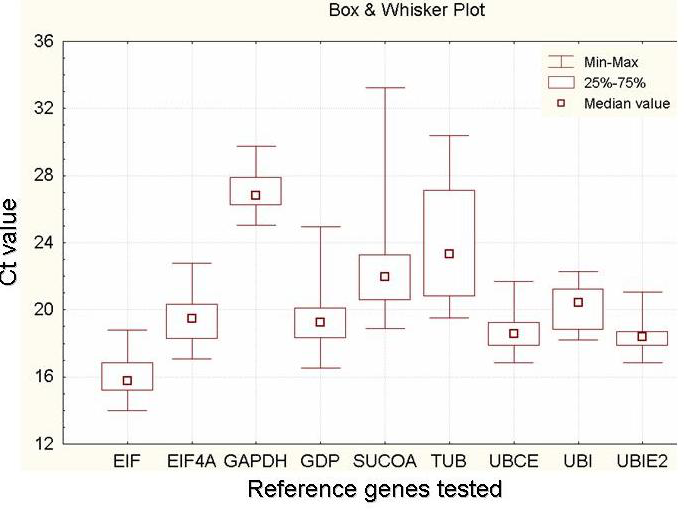
**Box-whisker showing the Ct variation of each candidate reference gene among the different tissue samples**. The median quartiles and the minimum and maximum Ct of the 22 samples were calculated in the Statistica program.

**Figure 2:**
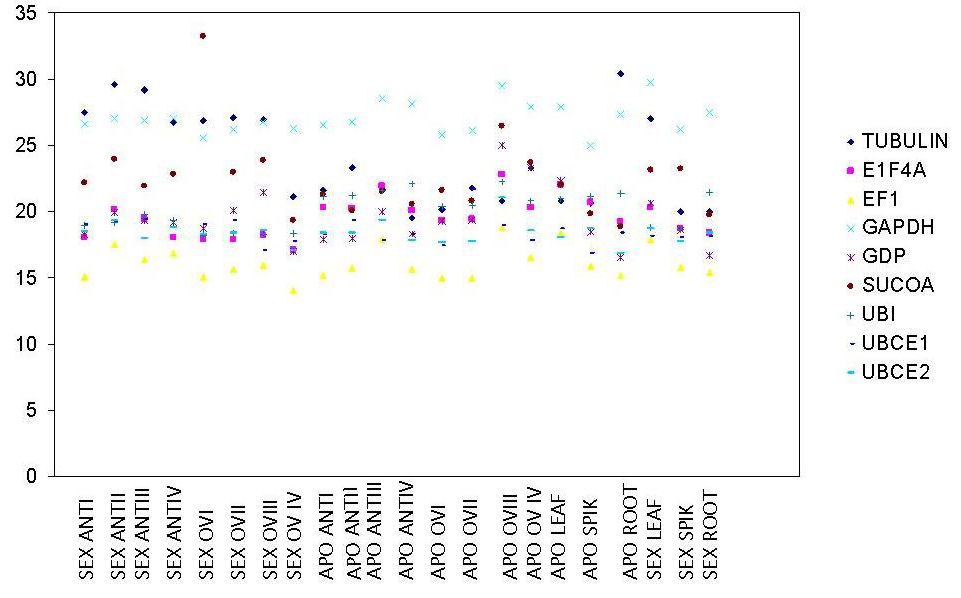
**Ct distribution of each candidate reference gene among the 22 samples**. Sex: cDNA from BRA 002747; Apo: cDNA from BRA 00591; ANT: anthers; OV: ovaries; SPIK: spikelet; I–II: sporogenesis; III–IV: gametogenesis.

The median Ct data in the 22 samples are shown in Figure 1, and the Ct variations among the samples for the different primers are shown in Figure 2. The range and distribution of the Ct values allow for a visualization of the least variable genes among the samples. This provides an indication of the most stable genes, which were ubiquitin-conjugating enzymes (*Bbriz*UBCE1 and *Bbriz*UBCE2) and elongation factor-1 alpha (*Bbriz*EF1), and showed the narrowest Ct range and therefore the least deviation from the Ct median by the different samples (Figure 1). The Ct values of the candidate reference genes in all samples were within 13.99 and 33.22 cycles, showing a high range of variation between them. *Bbriz*EF1 showed the highest expression levels, whereas *Bbriz*SUCOA showed the lowest within the chosen set of samples. Depending on the expression level of the genes tested, it is suitable to choose a reference gene with similar expression levels of the tested genes.

### Gene expression stability of candidate reference genes

**Figure 3:**
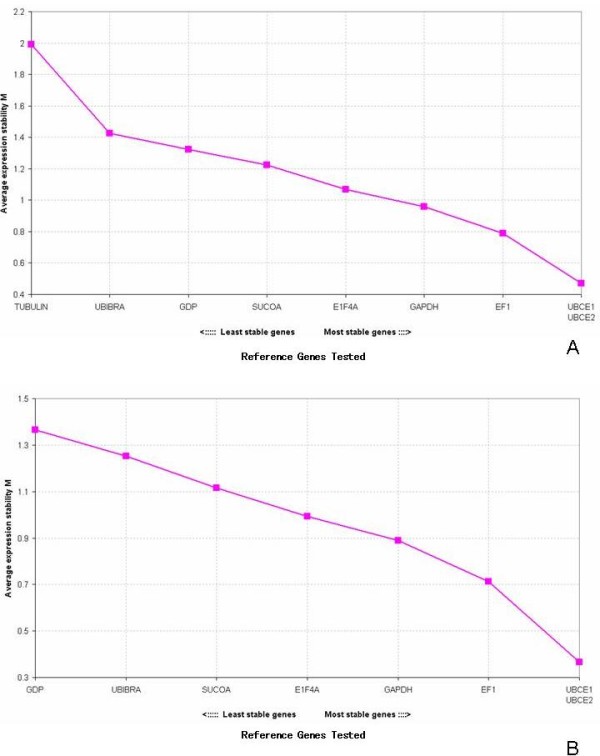
**Average expression stability values (M) of the control reference genes using geNorm**. Plotted from the least stable to the most stable. **A**: including all 8 genes and 9 primer pairs. **B**: excluding the least stable gene, *Bbriz*TUB.

We used the geNorm application for selecting the best reference gene for *Brachiaria brizantha *[17]. GeNorm calculates a gene stability value (M) and a normalization factor (NF) based on the geometric mean of the expression values of the set of the control genes tested. The lower the M value, the more stably expressed the gene is. Also, the program enables the exclusion of the most unstable gene to recalculate the M value. Out of the eight genes used for analysis, only *Bbriz*TUB showed an M value higher than the cutoff established by geNorm (M < 1.5), and two of them (ubiquitin-conjugating enzyme and elongation factor-1 alpha) showed the lowest M values, numbers well-suited for reference genes [27,28]. The primers used for the ubiquitin-conjugating enzyme (*Bbriz*UBCE1 and *Bbriz*UBCE2) and elongation factor-1 alpha (*Bbriz*EF1) had M values of 0.47 and 0.79, respectively, when all of the genes for the calculation were considered (Figure 3a). However, after exclusion of the least stable gene, *Bbriz*TUB, there was a decrease in the M value of all the other genes and also a change in the M values of the unstable genes, *Bbriz*GDP and *Bbriz*UBI (Figure 3b). To check if the primer design might interfere in the stability value of gene expression and to have an internal control of geNorm, a second pair of primers was used for the ubiquitin-conjugating enzyme that amplified a more external region of the gene. The M value for both sets of primers was the same, showing that the amplification region had no influence on the expression stability. Although the difference in the M value of the *Bbriz*EF1 gene was higher when compared with *Bbriz*UBCE, it is recommended its inclusion as a reference gene in qRT-PCR because of its high expression values, which is important whenever the tested genes are highly expressed. To support these data, Bestkeeper [19], another Excel-based tool based on pairwise correlation, was performed and showed similar results concerning the best reference genes for *Brachiaria *(data not shown). Having at least two reference genes is suggested for a more accurate qRT-PCR analysis. A previous report concerning the reference genes for *Oryza sativa *also showed that ubiquitin 5 and EF1 were the most stable genes for the tissues analyzed [21]. In addition, a recent work on *Vitis vinifera *identified elongation factor-1 as one of the most stable genes for pre- and post-anthesis flowers, berries, leaves and roots [22]. In species that show both apomictic and sexual development, differential expression screening in immature spikelets have been held in order to find genes related to apomixis development. For *Eragrostis curvula *it has been shown that, depending on the ploidy level and reproductive development of the plant, genes that are usually constitutive, such as ubiquitin and elongation factor can vary in expression level [25]. In addition, for *Paspalum notatum*, another apomictic plant, among other genes, polyubiquitin and ribossomal protein showed different expression levels depending on the ploidy and reproductive development when comparing immature spikelets of apomictic vs sexual plants [24]. Considering that in these two species the reproductive mode and ploidy level influence in expression levels of commonly used reference genes and if they will be used for apomixis molecular studies, stability in ovaries of sexual and apomictic plants is probably a relevant point to be considered. Therefore, the M value for only spikelets and ovary tissues in the four developmental stages in both apomictic and sexual *B. brizantha *was calculated (additional file 2). *Bbriz*UBCE and *Bbriz*TUB were again the more stable and the least stable genes respectively, while there was a slight difference in the order of stability of the other genes. The second and third best reference genes were *Bbriz*E1F4A and *Bbriz*EF1 with a 0.08 difference in M value.

**Figure 4:**
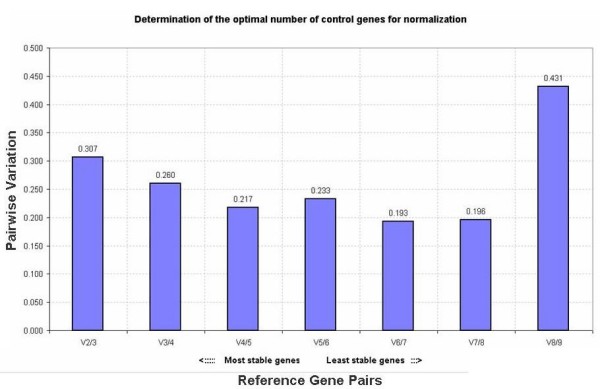
**Pairwise variation (V) of the selected reference genes**. Calculated on geNorm, from the most stable gene to the least stable according to the M value; V2/3 pairwise variation between the two most stable genes (UBCE1 and UBCE2) + 3^rd ^most stable gene (EF1); V3/4: addition of the 4^th ^most stable gene (GAPDH); V4/5: addition of the 5^th ^most stable gene (E1F4); V5/6: addition of the 6^th ^most stable gene (SUCOA);. V6/7: addition of the 7^th ^most stable gene (GDP); V7/8: addition of the 8^th ^most stable gene (UBIBRA); V8/9: addition of the 9^th ^most stable gene (TUB).

The geNorm application considers two different factors in order to analyze gene expression stability: the average expression stability (M) and the pairwise variation (V). The pairwise variation (V_n/n+1_) is calculated based on normalization factor values after the stepwise addition of a least stable reference gene (NF_n _and NF_n+1_) and indicates the minimum number of reference genes necessary for an accurate normalization. When analyzing all eight of the genes with a pairwise variation, there was not a significant difference in the V numbers, but there was an increase in the instability with the addition of *Bbriz*SUCOA (V5/6) and *Bbriz*TUB (V8/9, Figure 4), relatively unstable genes as shown by the Ct distribution in Figures 1 and 2. The optimal cutoff V number according to Vandersompele (2002) should be around 0.15, but other works using this application have shown a higher V number for the studied species [29,30], depending on the amount of genes and type of samples tested. In *B. brizantha*, the addition of a fourth gene did not have a significant contribution to stability, comparing all tissues. Considering these values, we suggest that only two reference genes, *Bbriz*UBCE and *Bbriz*EF1, should be used for qRT-PCR experiments of root, leaf and reproductive tissues of the studied accessions. Besides, analysis of ovaries alone should be performed preferentially with *Bbriz*UBCE, *Bbriz*E1F4 and *Bbriz*E1F.

## Conclusion

From the eight housekeeping genes tested in this study, the ones encoding for the ubiquitin-conjugating enzyme (*Bbriz*UBCE) and elongation factor-1 (*Bbriz*EF1) were considered most stable based on the transcriptional profile and geNORM analysis when considering both vegetative and reproductive tissues.

These two genes have been suggested as reference genes in other plants for qRT-PCR analysis, but also for other experimental techniques such as RT-PCR and northern blot analysis [21,31,32]. Even though the two genes exhibited the desired stability values, the best experimental designs use reference genes that act independently and are involved in different cellular processes. Therefore, *Bbriz*UBCE and *Bbriz*EF1 will be used as the reference genes in further experiments of *B. brizantha *vegetative and reproductive developmental tissues. This is the first report to clone, sequence and test reference genes for the transcriptional analysis of plants of the *Brachiaria *genus. Our results provide crucial information for transcriptional analysis in the *Brachiaria *ssp, helping to improve the quality of gene expression data in these species, which constitute an excellent plant system for the study of apomixis.

## Methods

### Plant material and tissue samples

Two accessions of *Brachiaria brizantha *from Embrapa were used in this work: BRA 002747 (B105), a sexual diploid (2n = 2x = 18), and BRA 00591 (B030), a facultative apomictic tetraploid (2n = 4x = 36) named *B. brizantha *(A. Rich) Stapf cv. Marandu, which were both cultivated in the field at the Embrapa Genetic Resources and Biotechnology.

For analysis of the most stable genes during male and female gametophyte development, ovaries and anthers of both accessions were dissected from flowers at four different stages of development before anthesis, as previously described by Rodrigues et al. (2003). For each RNA extraction experiment, around 1000 ovaries and 50 anthers of each of the four stages were isolated. Stages I and II are related to sporogenesis, and stages III and IV are related to gametogenesis [9,6]. In addition, whole spikelet, leaf and root tissues were isolated from both *B. brizantha *accessions for RNA extraction.

### RNA extraction

Total RNA was extracted from each pool sample with TRIZOL (1/10 w/v) (Invitrogen™) with a modified method from the manufacturer's instructions. Samples were ground with a drill (AD-18 S Bionic Drill set) holding an RNAse-free polystyrene pistil. After extraction, the RNA sample was dissolved in 15–20 *μ*l of 0.1% diethyl pyrocarbonate (DEPC)-treated water. RNA was treated with DNAse using on-column Qiagen DNAse Treatment (RNeasy MicroKit, Qiagen, Valencia, CA, USA) according to the manufacturer's instructions. The RNA concentration and A_260_/A_280 _ratios were determined before and after DNAse I treatment using a Nano-Drop ND-1000 spectrophotometer (NanoDrop Technologies), and 1.1% agarose gel electrophoresis was conducted to visualize the integrity of the RNA. Only the RNA samples with A_260_/A_280 _ratios between 1.9 and 2.1 and A_260_/A_230 _ratios greater than 2.0 were used for the analysis.

First strand cDNAs were synthesized from 1.5 μg of total RNA with OligodT and Superscript II enzyme (Invitrogen™). The first strand synthesis system was used according to the manufacturer's instructions.

### PCR primer design

Several described plant housekeeping genes were selected for the analysis. Genes already described as good reference genes for other plant species were used to BLAST search against *B. brizantha *EST (expressed sequence tag) ovaries libraries, and the list of selected sequences is shown in Table 1. Primers were designed within 800 bp of the polyadenylation site, since the primers came from an EST library constructed using the OligodT priming strategy. Primer 3.0 software was used for primer design. Amplicon lengths varied from 100 to 200 bp, with melting temperatures (Tm) varying between 59 – 60°C and primer lengths between 20–23 bp. The primers were screened for hairpins, dimmer formation, and target specificity by BLASTN  against the nr databank. Primer pairs were tested for specificity by RT-PCR and also by qRT-PCR, followed by a dissociation curve and agarose gel electrophoresis.

### Real-time PCR conditions and analysis

PCR reactions were performed in 96-well plates with the Chromo4 Real-Time PCR Detector System (BioRad^®^) using SYBR^® ^Green to detect dsDNA synthesis. Reactions were done in 20 μL volumes containing PCR Buffer (Invitrogen™), 1.5 mM MgCl_2_, 0.1 mM dNTPs, 0.25 U Taq Platinum (Invitrogen™), 0.1× SYBR Green (Amersham™), 200 nM of each primer and 10 μl sscDNA (corresponding to 5 ng of total RNA). Aliquots from the same sscDNA sample were used with all primer sets in two separate experiments. Two biological replicates for each of the 20 samples were used for real-time PCR analysis, and three technical replicates were analyzed for each biological replicate.

Reactions were run in a BioRad qRT-PCR machine using the following cycling parameters: 94°C for 5 min, 40 cycles of 94°C for 15 s, 60°C for 10 s, 72°C for 15 s and 60°C for 35 s. No-template controls (NTC) were included for each primer pair, and each PCR reaction was performed in triplicate. Dissociation curves for each amplicon and agarose gel were then analyzed to verify the specificity of each amplification reaction; the dissociation curve was obtained by heating the amplicon from 40°C to 100°C and reading at each °C.

### Primer efficiency calculation and Ct determination

The calculation of primer amplification efficiency and Ct determination were done using the miner algorithm [26]. Raw fluorescence data generated with the Opticon 3 software (BioRad) was used for these calculations. After running the miner algorithm, Ct values were transferred as a Microsoft Excel file (Microsoft, Redmond, WA) for further gene expression stability analysis.

### Analysis of gene expression stability

For analysis of gene expression stability and rank, geNorm v. 3.4 software was used. The Microsoft Excel file (Microsoft, Redmond, WA) with the raw expression Ct values for each tested gene in the 22 different samples generated with the miner algorithm was first analyzed with the qBase software version 1.3.4  and then transferred into the expression stability program geNorm, version 3.4 , as described by Vandesompele et al. (2002). This application defines the most stable genes by calculating the mean pairwise variation between a particular gene and all the others used in one experiment and determines an M value. The highest M value corresponds to the least stable expression in a set of samples. As a result, the normalization factor (NF) is defined, by considering the M value of the most stable genes. This information allows for the establishment of the minimum number of reference genes required for an accurate calculation of the relative expression of a target gene. This ideal number is given by the inclusion of a certain number of genes in the NF calculation until there is no significant contribution to an additional gene. The raw data from the two biological replicas was used for gene stability analysis and both showed similar results.

## Authors' contributions

EDS was responsible for the experiments, RNA sample preparation, qRT-PCR assays, data analysis and drafting the manuscript. LAG contributed with tissue isolation, RNA and cDNA sample preparation. FRS contributed on the bioinformatics analysis of the sequences tested. MAF and VTC participated as supervisors in the study design, analyses and writing. All authors contributed in writing the manuscript. All authors read and approved the final manuscript.

## Supplementary Material

Additional file 1 **Dissociation curves of the nine amplicons after the qRT-PCR reactions showing one peak for all of the technical replicas of all of the tested samples.** Arrows show no template control replicas. Click here for file

Additional file 2 **Average expression stability values (M) of the control reference genes using geNorm, plotted from the least stable to the most stable, using spikelets and ovaries in four developmental stages of sexual and apomictic accessions.** Click here for file
