# Supplementary figures and images for: Selection of reference genes for quantitative real-time PCR expression studies in the apomictic and sexual grass Brachiaria brizantha
Source: BMC Plant Biol. 2009 Jul 2;9:84. doi: 10.1186/1471-2229-9-84 (PMC2717968; doi:10.1186/1471-2229-9-84)

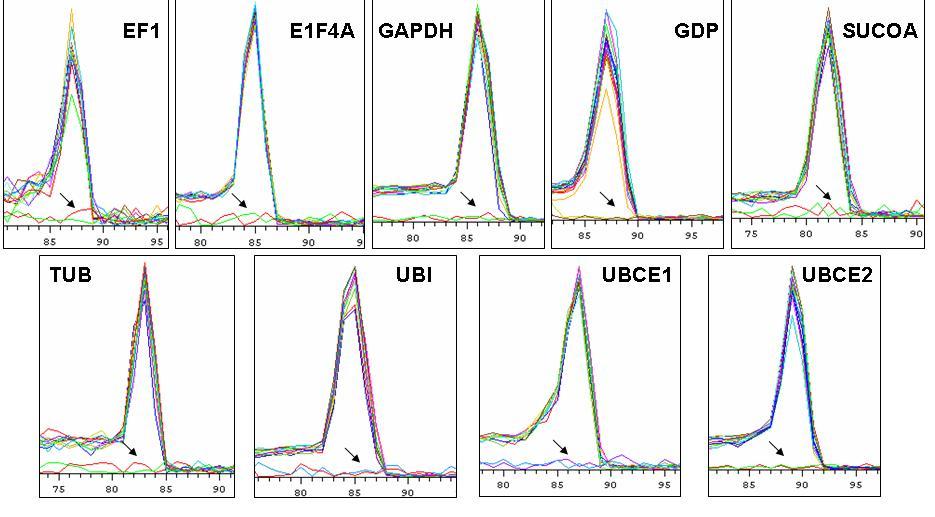

Supplement: Additional file 1 — Dissociation curves of the nine amplicons after the qRT-PCR reactions showing one peak for all of the technical replicas of all of the tested samples. Arrows show no template control replicas. [file 1471-2229-9-84-S1.jpeg]

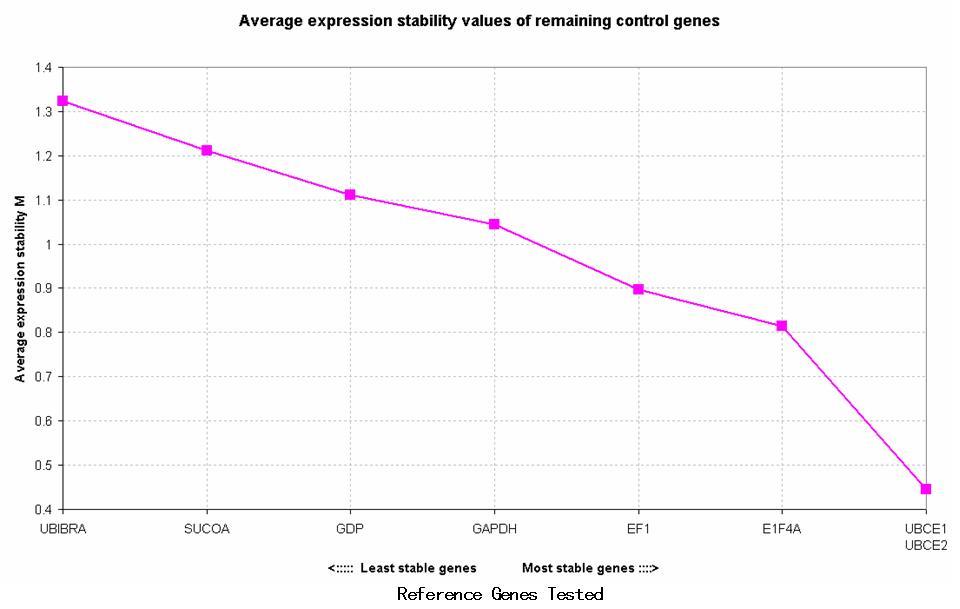

Supplement: Additional file 2 — Average expression stability values (M) of the control reference genes using geNorm, plotted from the least stable to the most stable, using spikelets and ovaries in four developmental stages of sexual and apomictic accessions. [file 1471-2229-9-84-S2.jpeg]
